# Supplementary material for: Opsonic Activity of Conservative Versus Variable Regions of the Group A Streptococcus M Protein
Source: Vaccines (Basel). 2020 May 7;8(2):210. doi: 10.3390/vaccines8020210 (PMC7349123; doi:10.3390/vaccines8020210)
Supplement: Supplementary file 1 [file vaccines-08-00210-s001.pdf]

vaccines

Supporting Information

Opsonic activity of conservative versus variable regions of the group A *Streptococcus* M protein

Chuankai Dai[1], Zeinab G. Khalil[2], Waleed M. Hussein[1,3], Jieru Yang[1], Xiumin Wang[1,4,5], Lili Zhao[1], Robert J. Capon[2], Istvan Toth[1,2,6] and Rachel J. Stephenson[1,*]

[1] School of Chemistry and Molecular Biosciences, The University of Queensland, St. Lucia, QLD 4072, Australia.

[2] Institute for Molecular Bioscience, The University of Queensland, St. Lucia, QLD 4072, Australia.

[3] Pharmaceutical Organic Chemistry Department, Faculty of Pharmacy, Helwan University, Helwan 11795, Egypt.

[4] Gene Engineering Laboratory, Feed Research Institute, Chinese Academy of Agricultural Sciences, Beijing 100081, People's Republic of China.

[5] Key Laboratory of Feed Biotechnology, Ministry of Agriculture and Rural Affairs, Beijing 100081, People's Republic of China.

[6] School of Pharmacy, The University of Queensland, Woolloongabba, QLD 4102, Australia.

[*] Correspondence: r.stephenson@uq.edu.au

Received: date; Accepted: date; Published: date

Table of Contents:

Analytical RP-HPLC and ESI-MS spectra of **VC-1**……………………………………………..Page S2

Analytical RP-HPLC and ESI-MS spectra of **VC-2**……………………………………………..Page S3

Analytical RP-HPLC and ESI-MS spectra of **VC-3**……………………………………………..Page S4

DLS spectra of **VC-1** and **VC-2**…………………………………………………………………..Page S5

TEM of **VC-1** and **VC-2**…………………………………………………………………………...Page S6

VC-1

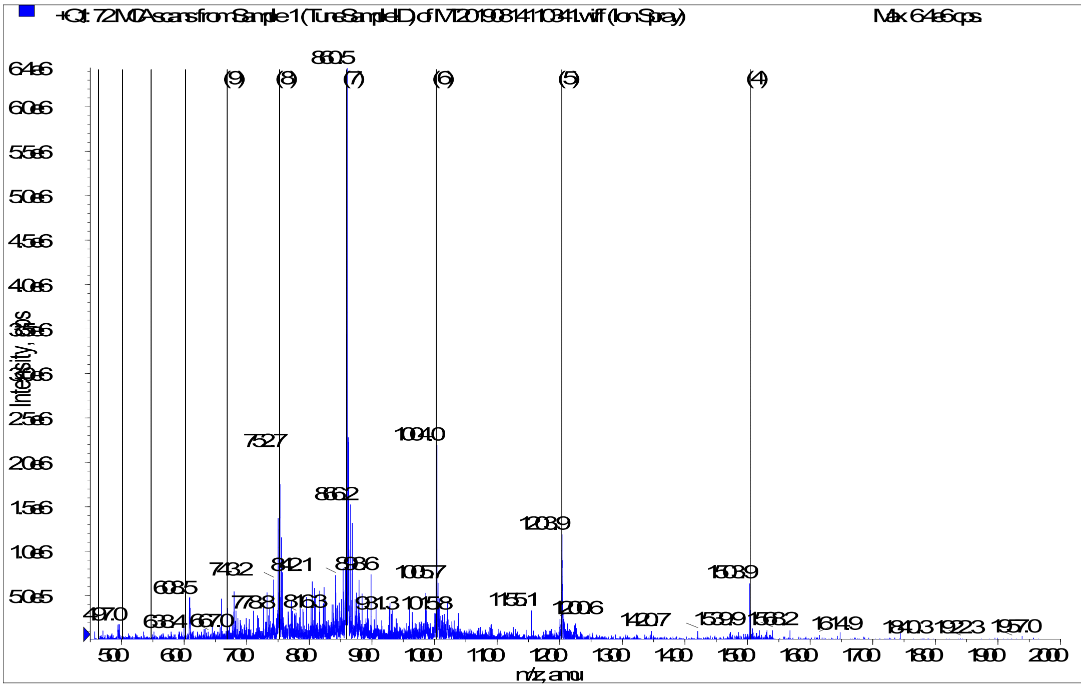

Figure S1: ESI-MS spectrum of VC-1. MW: 6014.2 Da. $[M+4H]^{4+}$=1503.9 (calculated 1504.6); $[M+5H]^{5+}$=1203.9 (calculated 1203.8); $[M+6H]^{6+}$=1004.0 (calculated 1003.4); $[M+7H]^{7+}$=860.5 (calculated 860.2); $[M+8H]^{8+}$=752.7 (calculated 752.8).

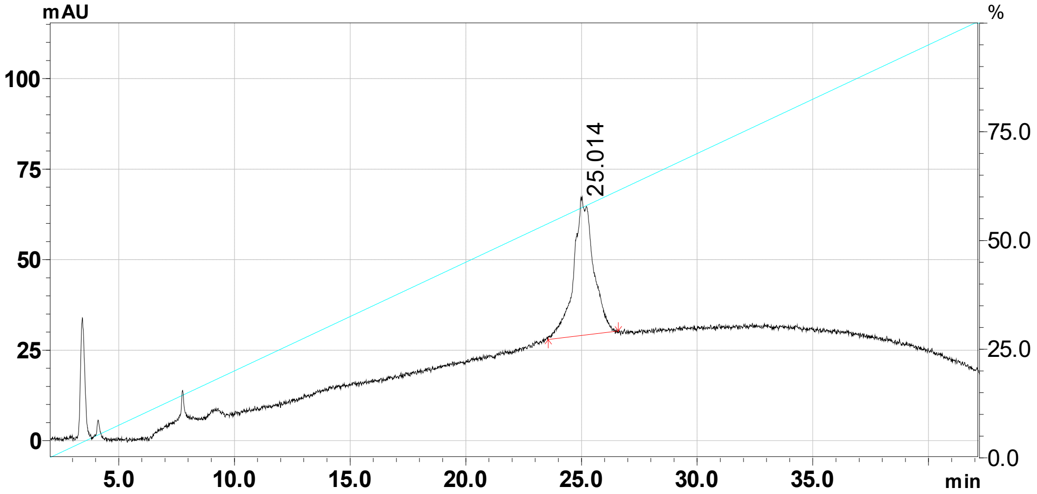

Figure S2: Analytical RP-HPLC spectrum of VC-1 (C4 column). T_R= 25.0 min.

VC-2

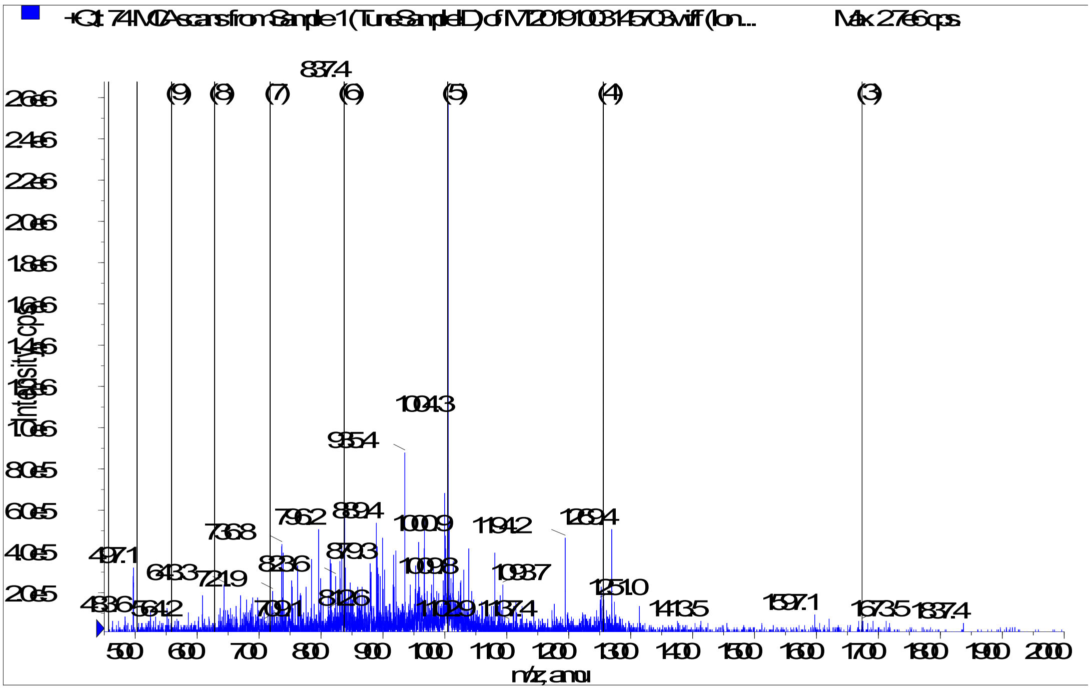

Figure S3: ESI-MS spectrum of **VC-2.** MW: 5018.0 Da.; $[M+3H]^{3+}$=1673.5 (calculated 1673.7); $[M+4H]^{4+}$=1255.4 (calculated 1255.5); $[M+5H]^{5+}$=1004.3 (calculated 1004.6); $[M+6H]^{6+}$=837.4 (calculated 837.3).

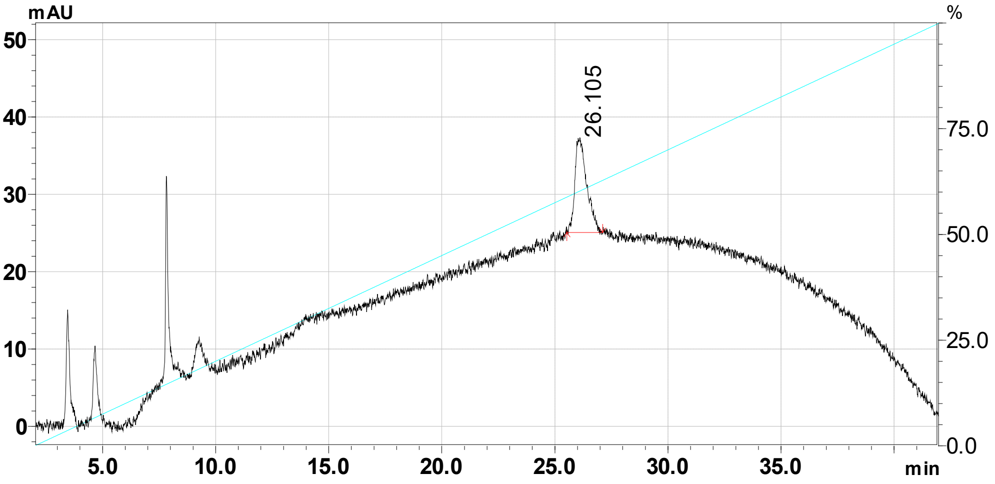

Figure S4: Analytical RP-HPLC spectrum of **VC-2** (C4 column). T_R= 26.1 min.

VC-3

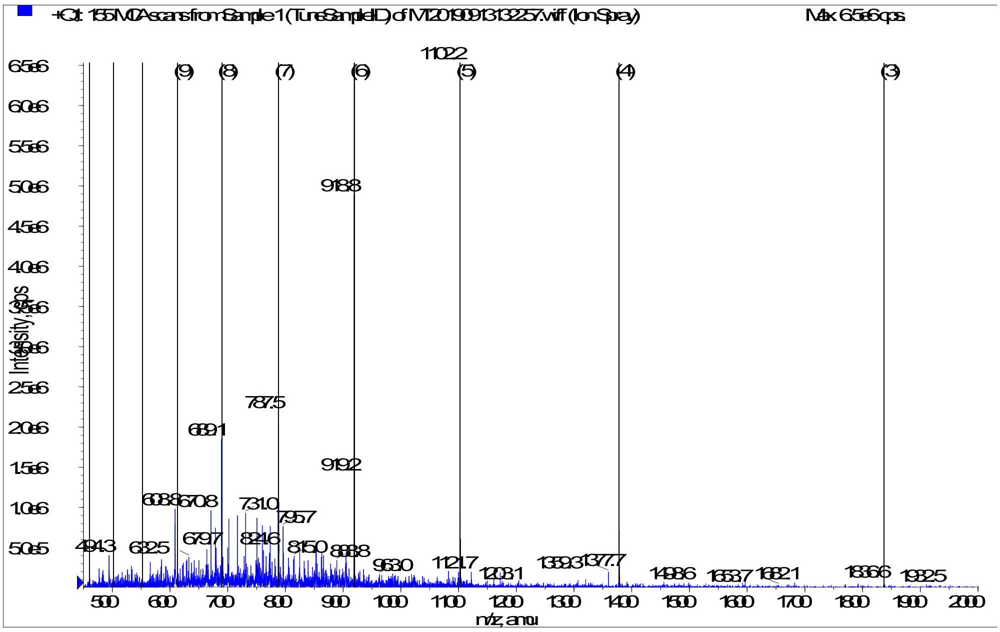

Figure S5: ESI-MS spectrum of **VC-3**. MW: 5507.4 Da.; $[M+4H]^{4+}$=1377.7 (calculated 1377.9); $[M+5H]^{5+}$=1102.2 (calculated 1102.5); $[M+6H]^{6+}$=918.8 (calculated 918.9); $[M+7H]^{7+}$=787.5 (calculated 787.8); $[M+8H]^{8+}$=689.1 (calculated 689.3).

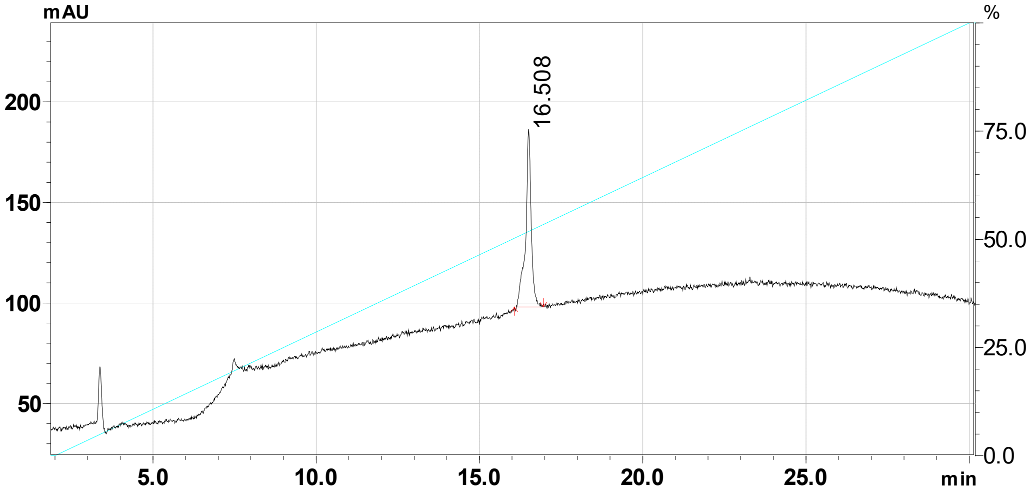

Figure S6: Analytical RP-HPLC spectrum of **VC-3** (C18 column). T_R= 16.5 min.

DLS spectra

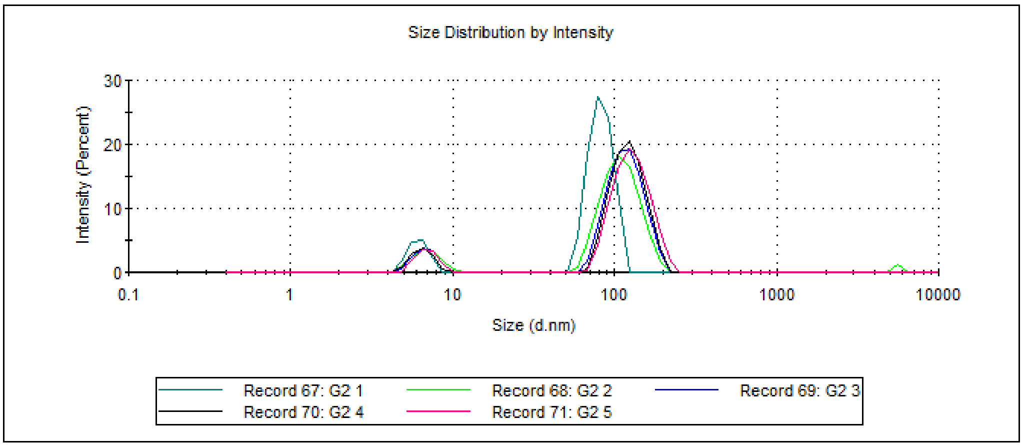

Figure S7: DLS spectra of particle **VC-1** size distributions by intensity.

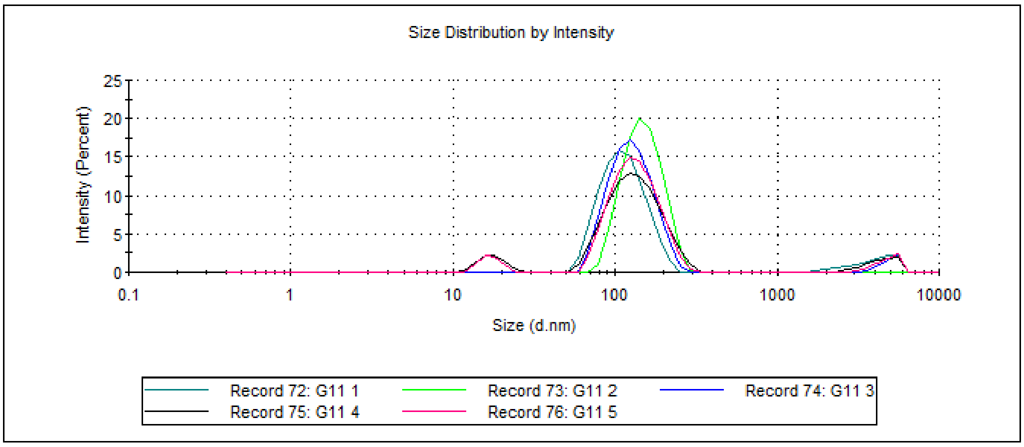

Figure S8: DLS spectra of particle **VC-2** size distributions by intensity.

TEM Images

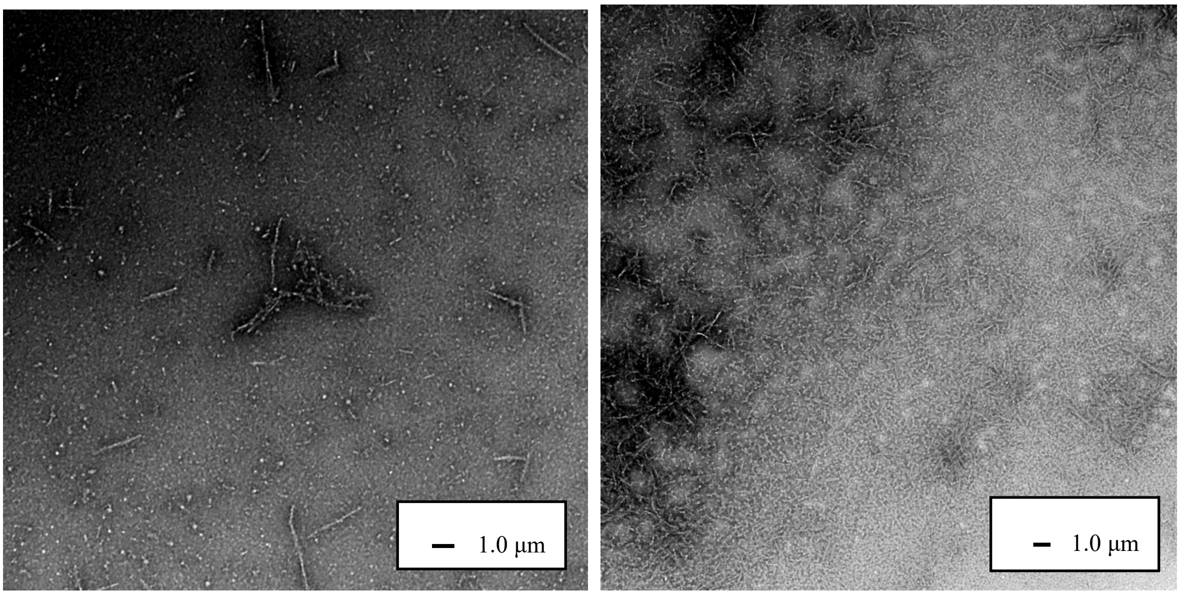

Figure S9: Left: Transmission electron micrograph of **VC-1** stained with 2% uranyl acetate.

Right: Transmission electron micrograph of **VC-2** stained with 2% uranyl acetate.
